# Chronic Fibrosis and Its Progression to Cancer

**DOI:** 10.3390/ijms23073924

**Published:** 2022-04-01

**Authors:** Taro Yasuma, Esteban C. Gabazza

**Affiliations:** Department of Immunology, Mie University Faculty and Graduate School of Medicine, Tsu 514-8507, Mie, Japan; t-yasuma0630@clin.medic.mie-u.ac.jp

The terminal stage of many chronic inflammatory diseases is organ fibrosis. Fibrogenesis is commonly associated with progressive organ scarring with decreased or loss of function [1]. Fibrosis of parenchymal organs is characterized by abnormally enhanced tissue deposition of components of the extracellular matrix, including several types of collagens, fibronectin, tenascins, and periostin [1]. The fibrotic process is generally reversible at the early stages, but it may become irreversible at later stages when the tissue injury is very severe or becomes chronic or repetitive [2]. Any organ can develop scarring tissue associated with different pathological states, including chronic intractable inflammation, infection, metabolic disorders, autoimmune disorders, graft rejection, or tumorigenesis [2].

Epidemiological and preclinical studies have shown that lifestyle, including dietary habits, is also implicated in fibrosis development of some organs, such as the liver [3]. Dietary habits are particularly important in subjects with non-alcoholic fatty liver disease (NAFLD) or chronic viral hepatitis. Patients with these underlying pathological conditions consuming cholesterol-rich food are prone to develop liver cirrhosis [4,5]. A diet enriched in cholesterol, saturated fatty acids, or trans fatty acids may promote liver fibrogenesis through several mechanisms by affecting the function of hepatocytes, Kupffer cells, hepatic stellate cells, or liver sinusoidal endothelial cells [3]. For example, in hepatocytes, the accumulation of free cholesterol in the endoplasmic reticulum triggers hepatocyte stress leading to cell injury, apoptosis, and fibrogenesis [3,6]. In Kupffer cells, increased uptake of cholesterol-rich low-density lipoprotein leads to the formation of foam cells, which activate hepatic stellate cells by stimulating the secretion of inflammatory chemokines and fibrotic cytokines [3].

NAFLD includes non-alcoholic fatty liver (NAFL) and non-alcoholic steatohepatitis (NASH) [7]. NAFL is characterized by hepatic steatosis without liver injury and NASH by the presence of inflammatory cells hepatocellular damage with or without fibrosis. NAFL may progress to NASH, and this to cirrhosis and hepatocellular carcinoma [8]. NASH is commonly associated with metabolic syndrome, obesity, diabetes mellitus, insulin resistance, and dyslipidemias [7]. Accumulating evidence has shown that enhanced inflammatory and immune responses, activation of pro-apoptotic pathways, and dysbiosis of the gut microbiome play critical roles in the pathogenesis of NASH [7]. The physiological implication of the gut microbiome dysbiosis has recently been the focus of many investigations. NASH patients showed an increased prevalence of small intestinal bacterial overgrowth, intestinal mucosa barrier malfunction, a low percentage of Bacteroidetes, and an abundance of Gram-negative bacteria in the gut microbiome [7]. In addition, microbial dysbiosis may increase the susceptibility to NASH, obesity, and type 2 diabetes mellitus [9].

NAFLD-associated hepatocellular carcinoma is also on the rise. NAFLD accounts for 14.1% of cases of hepatocellular carcinoma in the United States of America, 11.3% in Japan, and 34.8% in England [7]. Ethnicity, genetic/epigenetic factors, and aberrant activation of intracellular pathways (e.g., STAT3) have been implicated in the progression of NAFLD to hepatocellular carcinoma [7,10]. Importantly, the host immune response dysfunction contributes to the rapid progression of established hepatocellular carcinoma [11]. Tumor cells harness many mechanisms to evade the attack of the innate and acquired immune system. Induction of dendritic cells with high intracellular content of triglycerides that are unable to stimulate allogeneic T cells or to present tumor antigens, induction of tolerance to tumor antigens, secretion of immunosuppressive factors including interleukin (IL)-10 and transforming growth factor (TGF) β1 that increase the population of regulatory T cells, and expression of immune checkpoint inhibitors including programmed cell death ligand 1 (PD-L1) and PD-L2 that bind to the inhibitory receptor programmed cell death protein (PD-1) to inhibit the antitumor activity of CD8^+^ T cells [11].

The tolerogenic activity of myeloid-derived suppressor cells and regulatory T cells of the tumor microenvironment is another important factor favoring tumor evasion from the immune system attack [11,12]. In addition, the tumor microenvironment has an abundant population of cancer-associated fibroblasts (CAFs). The number of CAFs is a significant predictor of prognosis in many types of malignancy [13,14]. They may derive from activated resident fibroblasts, bone-marrow-derived mesenchymal stromal cells, and epithelial or endothelial cells after mesenchymal transition [13,14]. CAFs derive from activated hepatic stellate cells in hepatocellular carcinoma [15]. Cancer cells and surrounding activated normal cells secrete several growth factors, including TGFβ1, epithelial growth factor, vascular endothelial growth factor, tumor necrosis factorα, and IL-6 that promote CAF trans-differentiation [13,14]. CAFs promote proliferation, metastasis, and invasive behavior in many types of tumor cells by secreting growth factors and cytokines. Interestingly, a longitudinal evaluation of CAF development in mammary tumors identified a transcriptomic signature that allows one to distinguish CAFs from early and late-stage tumors, suggesting the plasticity of fibroblasts during cancer development [16]. The transcriptomic signature could predict the clinical stage and survival of human breast cancer patients [16].

The role of activated fibroblasts or cell-to-become CAFs in carcinogenesis has also been reported [17]. This observation suggests the potential implication of CAFs in the progression of chronic fibrosis to cancer. It is well-recognized that cancer frequently develops in fibrotic organs such as hepatocellular carcinoma in liver cirrhosis, lung cancer in idiopathic pulmonary fibrosis, and oral squamous cell carcinoma in oral submucous fibrosis [7,14,18]. For example, the potential malignant rate of oral submucous fibrosis to oral squamous cell carcinoma has been reported to be up to 30% [18,19]. Oral submucous fibrosis is associated with the habit of chewing betel quid or areca nut [18,20]. The mechanism of malignant transformation of oral submucous fibrosis is unclear. However, inflammatory (hypoxia-inducible factor1α, IL-6, IL-8) and profibrotic cytokines (e.g., TGFβ1), and the production of reactive oxygen species (ROS) appear to play critical roles [20,21,22]. Oxidation and nitrosation of areca nut components lead to the enhanced release of and prolonged cell exposure to ROS that cause DNA damage (e.g., DNA double-strand breaks) by oxidative stress [20,21,22]. In addition, increased inflammatory cytokines stimulate trans-differentiation of CAFs or senescent fibroblasts to induce epithelial-to-mesenchymal transition and promote cancer invasion and metastasis [20].

Advances in diagnostic techniques and therapeutic modalities have improved the life expectancy of many patients with chronic fibrosis and cancer. However, the prevalence of these incurable diseases is still increasing, and the mechanism of disease progression remains unclear. Therefore, there is an urgent need to elucidate the intricate molecular mechanisms that perpetuate the fibrotic process and trigger the progression of fibrosis to cancer for developing novel and more effective therapeutic approaches.
